# Supplementary material for: Medical Residents’ Behaviours toward Compulsory COVID-19 Vaccination in a Tertiary Hospital in Italy
Source: Int J Environ Res Public Health. 2022 Nov 30;19(23):15985. doi: 10.3390/ijerph192315985 (PMC9736902; doi:10.3390/ijerph192315985)
Supplement: Supplementary file 1 [file ijerph-19-15985-s001.zip › ijerph-2020273-supplementary.pdf]

International Journal of
Environmental Research and Public Health

MDPI

Supplementary material.

Table S1. Coding of Italian residencies in frontline and non-frontline in managing COVID-19 patients.

Code	Residency
COVID-19 frontline residencies	Anaesthesia and resuscitation;<br>Hygiene and preventive med *;<br>Diseases of the respiratory system;<br>Infectious diseases;<br>Emergency-urgent medicine;<br>Internal medicine
COVID-19 non-frontline residencies	Radiodiagnostics; Paediatrics; General surgery; Gynaecology and obstetrics; Orthopaedics and traumatology; Cardiovascular diseases; Geriatrics; Medical oncology; Psychiatry; Neurology; Endocrinology and metabolic diseases Child neuropsychiatry; Radiotherapy; Urology; Ophthalmology; Digestive system diseases; Neurosurgery; Otolaryngology; Haematology; Orthodontics; Forensic medicine; Nephrology; Occupational medicine; Physical and rehabilitative medicine; Dermatology and venereology; Vascular surgery; Rheumatology; Cardiac surgery; Sports and exercise medicine; Nuclear medicine; Pathological anatomy; Oral surgery; Medical genetics; Plastic, reconstructive and aesthetic surgery; Thoracic surgery; Medical physics; Allergology and clinical immunology; Clinical pathology and clinical biochemistry; Microbiology and virology; Audiology and phoniatrics; Food science

* Hygiene and Preventive Medicine residency was considered as "frontline", because MRs were mostly involved as vaccinating doctors in the COVID-19 vaccination campaign.
